# Current Treatment Approaches for Affective and Anxiety Disorders in Adults with Intellectual and Developmental Disabilities

**DOI:** 10.1192/j.eurpsy.2025.1552

**Published:** 2025-08-26

**Authors:** H. M. Nguyen, D. Valle, B. Carr

**Affiliations:** 1 University of Florida College of Medicine, Gainesville, FL, United States; 2 Psychiatry, University of Florida College of Medicine, Gainesville, FL, United States

## Abstract

**Introduction:**

Adults with intellectual and developmental disabilities (IDD) have higher rates of psychiatric disorders, such as depression, anxiety, and bipolar disorder, compared to the general population. Unique challenges, like cognitive impairments and communication barriers, require tailored treatments. This poster reviews strategies for managing these conditions in adults with IDD, focusing on adaptations in psychopharmacology and psychotherapy.

**Objectives:**

To outline treatment approaches for depression, anxiety, and bipolar disorder in adults with IDD, highlight limitations and necessary adaptations, and advocate for collaborative treatment models involving healthcare providers and caregivers.

**Methods:**

A literature review identified studies and guidelines on psychopharmacologic and psychotherapeutic interventions tailored to adults with IDD, examining the effectiveness of pharmacological agents, cognitive behavioral therapy (CBT), and other adaptations.

**Results:**

Current treatments for depression, anxiety, and bipolar disorder in adults with intellectual and developmental disabilities (IDD) often deviate from standard protocols, requiring modifications in both pharmacological and therapeutic approaches. Depression management in IDD typically relies on selective serotonin reuptake inhibitors (SSRIs), adapted with gradual dose escalation and close monitoring due to limited data on their specific effects in this population. Psychotherapy, particularly group cognitive behavioral therapy (CBT), has shown notable efficacy, with studies reporting significant symptom reduction in treated groups. For anxiety disorders, low-dose SSRIs remain the primary pharmacological option, with cautious titration to minimize adverse effects, while benzodiazepines are generally avoided to prevent paradoxical responses and disinhibition. CBT-based interventions, including graduated exposure therapy customized for specific phobias or triggers, show promise, though further randomized trials are warranted. Managing bipolar disorder in IDD is particularly challenging due to the heightened risk of severe functional impairment and symptom overlap, with mood stabilizers like lithium and antipsychotics administered sparingly given potential metabolic and neurological side effects. Given limited research, clinical strategies often rely on individualized treatment plans informed by provider expertise and patient-specific needs.

**Conclusions:**

Treatment for psychiatric disorders in adults with IDD requires significant adaptation, with careful dosing and monitoring of medications to minimize adverse effects. Evidence supports CBT as an effective option, yet there is a critical need for more research, especially randomized trials, to develop more robust guidelines specific to this population. Close collaboration between healthcare providers and caregivers is essential for successful outcomes.

**Disclosure of Interest:**

None Declared

